# A Review of Surrogate Assisted Multiobjective Evolutionary Algorithms

**DOI:** 10.1155/2016/9420460

**Published:** 2016-06-12

**Authors:** Alan Díaz-Manríquez, Gregorio Toscano, Jose Hugo Barron-Zambrano, Edgar Tello-Leal

**Affiliations:** ^1^Facultad de Ingeniería y Ciencias, Universidad Autónoma de Tamaulipas, 87000 Victoria, TAMPS, Mexico; ^2^Information Technology Laboratory, CINVESTAV-Tamaulipas, 87130 Victoria, TAMPS, Mexico

## Abstract

Multiobjective evolutionary algorithms have incorporated surrogate models in order to reduce the number of required evaluations to approximate the Pareto front of computationally expensive multiobjective optimization problems. Currently, few works have reviewed the state of the art in this topic. However, the existing reviews have focused on classifying the evolutionary multiobjective optimization algorithms with respect to the type of underlying surrogate model. In this paper, we center our focus on classifying multiobjective evolutionary algorithms with respect to their integration with surrogate models. This interaction has led us to classify similar approaches and identify advantages and disadvantages of each class.

## 1. Introduction

Multiobjective evolutionary algorithms (MOEAs) have been successfully applied to an important variety of difficult multiobjective optimization problems (MOPs) [1]. MOEAs are population-based techniques that make a multidimensional search, finding more than one solution within a single execution. Their main advantage lies in their ability to locate solutions close to the global optimum. However, in a number of real-world computationally expensive optimization problems, the number of calls of the objective function to locate a good solution can be high, even with these approaches. Moreover, in many science and engineering problems, researchers have used computer simulations in order to replace expensive physical experiments with the aim of improving the quality and performance of engineered products and devices, but using a fraction of the needed effort (e.g., computational fluid dynamics solvers, computational electromagnetics, and computational structural mechanics). However, such simulations are often computationally expensive, such that they can take several days or even weeks to be complete.

The use of surrogate models (we will use the terms approximation models, surrogate models, and metamodels interchangeably in this work) has been a recurrent approach adopted by the evolutionary computation community in order to reduce the fitness function evaluations required to produce acceptable results. Therefore, having a number of proposals that make use of surrogate models in MOEAs was predictable. Santana-Quintero et al. [2] and Landa-Becerra et al. [3] have reviewed independently such proposals, classifying them according to the type of surrogate model at hand (e.g., Kriging, radial basis functions, and polynomial regression). However, both works have shelved MOEA's point of view (i.e., how the surrogate model is incorporated into MOEA's evolutionary process). Jin [4] proposed a classification based on the way single-objective evolutionary algorithms (EAs) incorporate surrogate models. Shi and Rasheed [5] also adopted this kind of taxonomy calling it “working style classification.” According to such a classification, the approaches are divided into direct fitness replacement (DFR) methods and indirect fitness replacement (IFR) methods [5]. In the former group, the approximated fitness replaces the original fitness during the entire course of the EA process, while, in the latter group, some but not all processes (e.g., such as population initialization or EA operators) use the approximated fitness.

In this paper, we adopt the classification proposed by Jin [4] in order to undertake a classification with MOEA's point of view in mind. Therefore, the reviewed approaches are classified according to their working style. This type of classification allows finding easily similar works, however, placing greater emphasis on the methodology followed and not on the surrogate model used. Moreover, in this manner, it is possible to distinguish new opportunity areas according to each class in the taxonomy. The remainder of this paper is organized as follows.  Section 2 presents a brief overview of the surrogate modeling techniques commonly used in the literature.  Section 3 describes the adopted taxonomy to classify the surrogate-based multiobjective approaches reviewed in this paper. Sections 3.1 and 3.2 review surrogate-based approaches according to the adopted taxonomy. Finally, Section 4 summarizes our main conclusions about the existing works in the literature with respect to the use of surrogate models in MOEAs.

## 2. Surrogate Model Techniques

A surrogate model is an approximation of a simulation used to construct simpler and lower computational cost models; if the original simulation is represented as $f(\vec{x})$ and the metamodel is represented as $f^{\prime }(\vec{x})$, then, $f^{\prime }(\vec{x})=f(\vec{x})+e(\vec{x})$, where $e(\vec{x})$ is the approximated error. The internal behavior of $f(\vec{x})$ does not need to be known (or understood); only the input/output behavior is important. A model is constructed based on the response of the simulator to a limited number of intelligently chosen data points. Metamodels generate simpler representations that capture relations between the relevant information of the input and output variables and not in the underlying process.

Among the techniques to create surrogate models, we have rational functions [6], radial basis functions [7], Kriging models [8], support vector machines [9], polynomial regression [10], and splines [11]. Below, we review the most common approaches for constructing approximate models.

### 2.1. Polynomial Approximation Models

The response surface methodology (RSM) [10] employs statistical techniques for regression and analysis of variance in order to obtain a minimum variance of the responses.

The simplicity of polynomials makes them a good approach to approximate most polynomial response surfaces (PRS).

A polynomial in the coded inputs ${\vec{x}}_{1},{\vec{x}}_{2},\ldots ,{\vec{x}}_{n}$ (*n* data in the training set) is a function which is a linear aggregate (or combination) of powers and products of the input.

The polynomial model is usually written in matrix notation, ${\hat{y}}^{\left(p\right)}=\beta^{T}{\vec{x}}^{p}$, where *β* is the vector of coefficients to be estimated and ${\vec{x}}^{p}$ is the vector corresponding to the form of *x*_1_^(*p*)^ and *x*_2_^(*p*)^ terms in the polynomial model.

To estimate the unknown coefficients of the polynomial model, both the least-squares method (LSM) and the gradient method can be used. However, both approaches require the number of samples to be equal to the number of coefficients.

PRS can also be built using stepwise regression [12]. The basic procedure for stepwise regression involve (1) identifying an initial model, (2) iteratively “stepping,” that is, repeatedly altering the model at the previous step by adding or removing a predictor variable in accordance with the “stepping criterion,” and (3) terminating the search when a specified maximum number of steps has been reached.

### 2.2. Kriging-DACE

The response surface method called Kriging (KRG) [13] is a spatial prediction method that belongs to the group of geostatistical methods. It is based on minimizing the mean squared error, and it describes the spatial and temporal correlation among the values of an attribute.

The design and analysis of computer experiments (DACE) is a parametric regression model developed by Sacks et al. [8], which is an extension of the Kriging approach in order to be able to manage three or more dimensions.

The DACE model can be expressed as a combination of a known function $a(\vec{x})$ (e.g., polynomial function, trigonometric series) and a Gaussian random process $b(\vec{x})$ that is assumed to have mean zero and covariance:

(1) Ebx→i,bx→jCovbx→i,bx→j=σ2Rθ→,x→i,x→j,

where *σ* ^2^ is the process variance of the response and $ℛ(\vec{\theta },{\vec{x}}^{\left(i\right)},{\vec{x}}^{\left(j\right)})$ is the correlation function with parameters $\vec{\theta }$. Among the different types of correlation models we have exponential, Gaussian, linear, spherical, cubic, and splines models.

### 2.3. Radial Basis Function Network

The radial basis function (RBF) method was proposed by Hardy [7] in 1971. RBF is a real-value function whose value depends only on the distance from the input to the center of the neuron, so that $\phi (\vec{x})=\phi (\left\|\vec{x}\right\|)$, or alternatively on the distance from some other point *c*, called a center. Any function *ϕ* that satisfies the property $\phi (\vec{x})=\phi (\left\|\vec{x}\right\|)$ is a radial function. The norm is usually the Euclidean distance, although other distance functions can be used.

Typical choices for the RBF include linear, cubic, multiquadratic, or Gaussian functions.

RBF commonly has three layers: an input layer that incorporates an identity function, a hidden layer with nonlinear RBF activation functions, and a linear output layer. The output, *φ* : *ℝ* ^*n*^ → *ℝ*, of the network is thus $\varphi (\vec{x})=\sum_{i=1}^{N}w_{i}\phi (\left\|\vec{x}-{\vec{c}}_{i}\right\|)$.

In order to adapt the RBF network for a particular task, three parameters need to be fine-tuned: the weights *w*_*i*_, the center vector ${\vec{c}}_{i}$, and the RBF width parameters *β*_*i*_.

### 2.4. Support Vector Regression

Support vector machines (SVMs) draw inspiration from statistical learning theory [9]. SVM is a set of related supervised learning methods which analyzes data and recognizes patterns. SVM constructs a hyperplane or a set of hyperplanes in a high-dimensional space that can be used for classification, regression, or other tasks.

SVM maps its inputs to a larger space; however, the cross products may be computed easily in terms of the variables in the original space making the computational load reasonable. The cross products in larger spaces are defined in terms of a kernel function *K*(*x*, *y*), which can be selected to suit the problem.

Through the introduction of an alternative loss function, SVM can also be applied to regression problems (the SVM for a regression problem is known as a support vector regression (SVR)). The loss function must be modified to include a distance measure.

Consider the problem of approximating the set of data with the linear function shown in

(2) $$\begin{array}{c} f\left(\;x\;\right)=\langle \;w,x\;\rangle +b. \end{array}$$

The optimal regression function is given by the minimum of the function:

(3) $$\begin{array}{c} \phi \left(\;w,\xi \;\right)=\frac{1}{2}{\left\|\;w\;\right\|}^{2}+C\overset{n}{\underset{i=1}{\sum }}\left(\;\xi_{i}^{-}+\xi_{i}^{+}\;\right), \end{array}$$

where *C* is a prespecified value and *ξ* ^+^ and *ξ* ^−^ are slack variables representing the upper and lower constraints on the outputs of the system.

## 3. Taxonomy of Surrogate-Based MOEAs

 Shi and Rasheed [5] classified hybrid approaches (MOEAs and metamodels) into two main groups (according to their working style). The first group, called DFR methods, refers to all those approaches that having assessed the solutions in the surrogate model assume that the achieved fitness is comparable to that assessed by the real function. The second group is called IFR methods. Approaches of this group do not compare fitness assessed in the surrogate model with that obtained by the real function. They focus on returning *n* best solutions according to the surrogate model and assume that the host approach will evaluate such solutions in the real fitness function.

Although the use of DFR seems to be the most straightforward approach in using surrogate models, it should be used carefully since its behavior is highly dependent on the accuracy of the surrogate model, while the nature of the IFR accepts the surrogate model to be less precise.

DFR has three kinds of model management or evolution control, which refer to the way that the surrogate model deals with the real fitness function:

- *No Evolution Control (NEC)*. MOEAs that incorporate this approach evaluate their solutions in the surrogate model exclusively. However, the lack of feedback can mislead the obtained results. No evolution control should only be used when one is completely sure that the obtained metamodel behaves similarly to the real function; if not, the results can be far from real.
- *Fixed Evolution Control (FEC)*. In this approach, only some generations or some individuals are evaluated in the surrogate model while the remaining population is evaluated using the real test function. Since new solutions are evaluated in the real test function, it is possible to feed the surrogate model with such information in order to improve its accuracy. This model management is quite successful. However, its behavior strongly depends on the switchback parameter. Therefore, a poor parameter setting can produce bad results.
- *Adaptive Evolution Control (AEC)*. This approach avoids any possible poor tuning setting of the previous model through the use of an adaptive control that adjusts the number of solutions that will be evaluated in the surrogate model.

### 3.1. Direct Fitness Replacement (DFR) Methods

The use of surrogate models with evolutionary computation is not such straightforward work as one may expect. When a surrogate model is not properly selected, or it is constructed with a reduced-size training sample, or the sample is unevenly distributed, the constructed model will usually be inaccurate. Therefore, if the MOEA calculates the fitness of the population exclusively with the surrogate model, the entire approach will have more probabilities of converging to a false optimum (a false optimum in multiobjective optimization is a Pareto front of the surrogate model that does not correspond to the true Pareto front in the real function). For this reason, in most cases the surrogate model is used alternately with the original fitness function. This alternation can be defined as the evolution control. The DFR methods need to be subclassified according to their evolution control.

#### 3.1.1. No Evolution Control (NEC)

This approach is assumed to have a high-fidelity surrogate model, and, therefore, the original fitness function is not used at all during the evolutionary process:

(i) Choi et al. [14] proposed the use of Kriging models within the nondominated sorted genetic algorithm II (NSGA-II) [15] in order to design a low-boom supersonic business jet. Since each fitness function evaluation for this problem represents high computational cost, the authors proposed replacing completely the real fitness function with a surrogate model. Therefore, the Kriging model was created before the execution of the evolutionary approach. Then, NSGA-II was used to optimize the surrogate model. This proposal was used in a biobjective problem with 17 decision variables. Although the proposed approach found solutions close to the real Pareto front, it did not find any solution belonging to the true Pareto set.
(ii) Lian and Liou [16] proposed a hybrid algorithm to optimize a two-objective transonic compressor blade with 8 decision variables. This works as follows. First, the search space is sampled through the use of a hypercube sampling approach [17]. Next, with the sampled points a second-order polynomial regression is constructed. Then, a genetic algorithm (GA) is executed using the surrogate model until it detects a slowdown in the convergence rate. Finally, a sequential programming method is applied to the solutions obtained with the GA. In this work, the authors noticed that the surrogate model was accurate. Therefore, they decided to replace the real function with it. The results indicate successful hybridization.
(iii) Gonzalez et al. [18] proposed a framework that supports the design of unmanned aerial vehicles. The framework uses the hierarchical asynchronous parallel evolutionary algorithm (HAPEA) [19] to optimize the designs. The authors decided to replace the real functions with a Kriging surrogate model. Although HAPEA assessed good results for three biobjective problems with 53 decision variables, the produced results were not validated with respect to the true Pareto front.
(iv) Goel et al. [20] proposed the use of a modified NSGA-II that uses an unbounded-size archive. This approach builds a response surface model to approximate each objective function. After the authors tested the accuracy of the produced surrogate models, they noticed that the metamodel produced good predicted solutions. For this reason, the optimization problem was solved exclusively with the use of surrogate models. This approach was developed to solve a multiobjective liquid-rocket injector design problem with four variables. According to the authors, the Pareto front obtained with the response surface helped in the visualization of trade-offs among several designs.
(v) Liao et al. [21] proposed a multiobjective optimization approach for crash safety design of vehicles using a stepwise regression model. In this approach, a set of sample points was computed to construct the stepwise regression models. The authors showed that the accuracy of the surrogate model was adequate to be used as a replacement of the real objective function. However, it should be noted that the solved problem is a low-dimensional problem with only five variables. After the authors had validated the accuracy of their approach, they incorporated it into NSGA-II. The obtained results indicate successful hybridization.
(vi) Husain and Kim [22] combined NSGA-II with surrogate models to optimize a liquid flow microchannel heat sink with three decision variables. In the proposed methodology, experimental designs were evaluated through numerical simulation; then a surrogate model was constructed. Next, NSGA-II was used to obtain the global Pareto front of the surrogate model. After that, a refinement phase was taken applying sequential quadratic programming (SQP) with NSGA-II solutions as the initial guesses. To perform the local search, the first objective was optimized while the second objective was restated as an equality constraint. The local search was repeated for the second objective function by restating the first objective as an equality constraint. This process gives two new sets of solutions, which are then merged with NSGA-II solutions. From these solutions, the dominated solutions are discarded and the duplicated solutions are removed to achieve the global Pareto-optimal solutions. The authors compared the performance of polynomial regression, Kriging, and RBFs. Such a comparison was performed according to two performance measures, (1) the accuracy of the surrogate model technique and (2) the suitability to use the surrogate model technique into the MOEA; that is, the authors execute the surrogate model technique/MOEA and compared the found solutions against the Pareto-optimal solutions. According to the authors, the Kriging model was the most accurate approach for predicting Pareto-optimal solutions.

*Review*. The previously reviewed approaches work in two phases. The first phase aims to build the surrogate model, while, in the second phase, MOEA optimizes the surrogate model. Although those proposals were found to be computationally efficient and accurate, it is important to note their low dimensionality in both decision and objective space. Having a more challenging problem (e.g., with a bigger decision search space) could produce inaccurate surrogate models and, therefore, the MOEAs would produce unreliable solutions. Therefore, we suggest the use of no control evolution only with low-dimensional problems. Although most approaches incorporate nondominated sorting and crowding distance into a GA, we think there are other selection mechanisms (such as *ϵ*-dominance [23], indicator-based selection mechanisms [24, 25]) and search engines that have earned the opportunity to be tested (differential evolution [26, 27], particle swarm optimization [28–30], and simulated annealing [31, 32]).

#### 3.1.2. Fixed Evolution Control (FEC)

Approaches that undertake fixed evolution control alternate evaluations between the real fitness function and the surrogate model [4, 33]. Two different approaches of fixed evolution control can be distinguished: those that alternate evaluations at individual level, called individual-based controls, and those that do so at a generation level, called generation-based controls. In the first, a fixed number of individuals are evaluated in the surrogate model while the remainder are evaluated in the original function on each generation. The second approach alternates the evaluation of all the individuals in the original function during a fixed number of generations, and then the evaluation is assessed using the surrogate model for a fixed number of iterations:

(i) Nain and Deb [34] proposed building a coarse approximation of the original problem with an artificial neural network (ANN) that uses a set of initial solutions as a training dataset. In this proposal, a GA optimizes the approximation model. Since it is possible that the initial approximation model can be inaccurate, the algorithm reconstructs the model at each generation. In this way, this algorithm progressively transforms the model into a fine-grained approximation model. This approach solved a geometric biobjective curve fitting problem with 39 variables. The results showed that this approach could find similar nondominated fronts with about 68% of the evaluations needed if the exact problem was used.
(ii) D'Angelo and Minisci [35] proposed coupling a modified version of the multiobjective Parzen-based estimation of distribution (MOPED) algorithm [36] with the Kriging approach. This algorithm uses an individual-based control approach. The surrogate model is incorporated into the evaluation process. First, the approach evaluates the solution with the surrogate model. Next, the solutions are ordered according to the ranking method proposed in MOPED. Then, *n*_*v*_ solutions are chosen through uniform sampling from the best half of the population (according to the rank). If their distances to the rest of the elements are greater than a parameter dist_min_, then they are added to the dataset and the surrogate model is updated. In this work, the algorithm is used to solve a biobjective optimization problem with five variables.
(iii) Voutchkov and Keane [37] proposed an approach that builds a different surrogate model for each objective function. Then, it executes NSGA-II using the built metamodels for several generations. The best solutions found by this procedure are evaluated in the real function; then the metamodel is reconstructed with these real-evaluated solutions. In this work, several response surface methods were compared according to their suitability to be incorporated into MOEA. The methodology was validated with three biobjective test problems. Two of these problems involve two decision variables and the third involves 25 decision variables. According to the authors, an advantage of this approach is that each objective could, in theory, be modeled by a different type of response surface method or some objectives that being cheap to compute will not need to be modeled at all. The results indicate that the Kriging metamodel performed well in most situations; however, it required much more computational time than the remaining metamodels. Moreover, the cubic spline RBFs produced acceptable results, besides combining low computational cost and having relatively fast convergence with respect to the other tested methods.
(iv) Isaacs et al. [38] proposed an algorithm that maintains an external archive with real function evaluated solutions. Data points in the external archive are split into multiple groups using *k*-means clustering. RBF is built for each cluster using a fraction of the points of such a group. The remaining points in each group are used as a validation dataset to decide the prediction accuracy of the surrogate model. The evaluation of a new solution is undertaken by the surrogate model that obtained the lowest prediction error in the neighborhood of the new solution. The proposed approach was compared to NSGA-II with a single global surrogate model on five low-dimensional (2 to 10 variables) biobjective test functions. The approach was tested with different numbers of clusters (3, 5, and 8). The results indicate that their proposal outperformed NSGA-II (with a single global surrogate model). In this proposal, the benefits of building local surrogates on smaller regions were clearly shown.
(v) Todoroki and Sekishiro [39] proposed a multiobjective framework to optimize a hat-stiffened composite panel with buckling constraint. This problem has one objective function and one constraint. The objective function is computationally cheap to compute. However, the constraint is computationally expensive. To solve this problem, the authors modified the original problem to a biobjective formulation with seven variables. The transformed problem maximizes the original objective function and the probability to satisfy the expensive constraint. Such a probability is obtained through the use of a Kriging surrogate model. The proposed approach uses the Multiobjective Genetic Algorithm (MOGA) [40] as the search engine. In this approach, MOGA's population is restarted after a fixed number of generations; then the Kriging model is updated with the ten individuals with the highest score. According to Todoroki and Sekishiro, their approach produced results using only 301 evaluations with a 3% error from the true optimal structure.
(vi) Liu et al. [41] proposed an approach that builds a quadratic polynomial regression every iteration; then a predicted Pareto-optimal set is identified with MOEA. Next, an approach to move the limits (lower bound and upper bound) of the variables is adopted in order to determine the motion limits of each design variable for the next iteration according to the predictions obtained with the surrogate model. The next surrogate model is constructed with the solutions that are in the region according to the limits of the design variables. The efficiency of this approach was validated in four test problems. Furthermore, it was evaluated on two engineering problems. According to the author's results, the proposed approach solved the test problems with about 10% of the evaluations with respect to the same approach but without a surrogate model. However, the results were tested in problems with only 2 and 3 variables.
(vii) Fonseca et al. [42] proposed a framework that uses similarity-based surrogate models. The core approach of such a framework harbors NSGA-II. This approach manages a repository to store the real objective solutions assessed so far. In every iteration, a surrogate model is constructed with solutions taken from such a repository. Then, NSGA-II is executed assessing its solutions in the surrogate model. Next, the approach selects the best solutions from NSGA-II's final population. These best solutions are selected according to the *psm* parameter, which represents the percentage of the population that will be selected. *psm* solutions are evaluated in the real objective function. This framework was tested in eight unconstrained and six constrained test problems with up to 30 variables. However, the results obtained by this approach were not compared to other MOEAs. According to the authors, small values of the *psm* parameter can help to achieve a better convergence to the true Pareto front.
(viii) Zapotecas Martínez and Coello Coello [43] proposed incorporating a cooperative surrogate model based on RBFs into the multiobjective evolutionary algorithm based on decomposition [44] (MOEA/D) in order to solve computationally expensive multiobjective problems. The proposed approach uses a subset of the evaluated solutions found by the MOEA/D to update both, the training dataset and the external archive. This algorithm was validated in five biobjective test problems with ten and 30 decision variables. The results showed that the proposed approach performed reasonably well for unimodal problems. However, in multimodal problems the algorithm had difficulties in obtaining a good representation of the true Pareto front. Additionally, this approach was used to solve an airfoil design problem with 12 variables. The results of this problem showed that the proposed approach is faster (requires less evaluations) than the original MOEA/D when the objective function is computationally expensive.
(ix) Stander [45] proposed an algorithm based on an adaptive domain reduction. In this approach, on each iteration NSGA-II is executed in a metamodel. From the obtained Pareto front, some solutions are selected to work as Pareto front kernels with a discrete space filling method (a strategy to generate good random points and achieve reasonably uniform coverage). Then, a subdomain is constructed on each kernel. For each subdomain, a continuous space filling procedure is performed in order to fill the subdomains with solutions that aim to improve the diversity. Finally, some points are selected to create a new surrogate model and, then, NSGA-II is reexecuted. This approach was tested in two biobjective problems, reaching the true Pareto front with only 2850 evaluations of the real objective function. Additionally, this approach was used to solve a full-vehicle multidisciplinary design optimization problem. According to the authors, in this specific problem with seven variables, the approach required only about 4% of the effort required by NSGA-II to obtain results with a similar accuracy, and for a 30-variable problem this number rises to about 15%.

*Review*. This approach can be seen as a natural evolution of the first category. In spite of its ease of implementation, this approach has the disadvantage of having to define the parameter to alternate between the surrogate model and the real function. Also, approaches that work under the fixed evolution control usually require more evaluations of the real objective function since those evaluations are used as feedback in order to improve the accuracy of the surrogate model. Additionally, it is easy to identify that most of the existing works in this category use NSGA-II as the underlying MOEA. However, there have been recent attempts to introduce newer MOEAs [43]. A main difference of the works in this category with respect to the works in the previous one is that these works were able to solve high-dimensional problems.

In spite of their success, most of these works did not include any studies of different surrogate models in order to select the most accurate for the problem at hand. Therefore, we think that proper selection of the surrogate models based on their accuracy can improve the general performance of these approaches.

#### 3.1.3. Adaptive Evolution Control (AEC)

Adaptive evolution control is similar to fixed evolution control. However, this approach adjusts the frequency of control according to one criterion (e.g., the accuracy of the surrogate):

(i) Gaspar-Cunha and Vieira [46] proposed an approach that alternates ANN to approximate the fitness of several benchmark problems with up to 30 variables and a polymer extrusion real-world problem with four variables; the ANN is used only when the estimated error of the neural network is lower than a predefined value. The results indicate that the proposed approach could reduce the computational time in the real-world problem by up to six hours.
(ii) Sreekanth and Datta [47] proposed a surrogate model based methodology to evolve multiobjective management strategies for saltwater intrusion in coastal aquifers with two objectives and seven variables. In this work, NSGA-II was combined with two different surrogate models: the first was based on genetic programming (GP-MOGA) while the second was based on a modular neural network (MNN-MOGA). In this approach, the surrogate models were constructed with patterns selected uniformly from the entire search space. Search space adaptation and model reconstructing were performed by identifying a modified search space near to the initial optimal solutions based on the relative importance of the variables in salinity prediction. If the salinity level predictions were not sufficiently accurate then the surrogate model was reconstructed using samples generated in the modified search space. On the downside in this work, the authors did not undertake any comparisons with other MOEAs.
(iii) Rosales-Perez et al. [48] proposed an approach that combines an evolutionary algorithm with an ensemble of surrogate models based on SVMs. In this approach, a set of surrogate models is constructed in order to determine, through the *k*-fold cross validation sampling technique, which surrogate model has the lowest expected generalization error. It is worth noting that each SVM is constructed with different parameters. This feature allows the algorithm to select the best surrogate model according to the area that is being explored in a particular generation of the MOEA. On each generation, new solutions are constructed using variation operators. Then, they are evaluated in the surrogate models. Next, the best solutions are stored in an external archive. If the archive is full, then its size is reduced with a clustering technique. When the estimations provided by the surrogate model are worse than a random solution, then the surrogates must be reconstructed. This approach was compared with NSGA-II in several biobjective test problems with ten and 30 variables. The results showed that this approach outperformed NSGA-II when both approaches carried out 3000 fitness function evaluations. However, the results also showed that the proposed approach had difficulties in converging to the true Pareto front of multifrontal problems.

*Review*. Unlike the previous category, approaches in this classification do not require the parameter to alternate between the real and the surrogate model evaluation. However, this automatic alternation approach is not easy to fine-tune. Most of the existing works use the error of the metamodel as the criterion to use the real objective function. However, a validation dataset is necessary to compute the error of the metamodel and sometimes it is difficult to have this dataset to measure the error.

The difficulty in gathering information in order to identify the signals for metamodel retraining precludes the proliferation of approaches in this category.

### 3.2. Indirect Fitness Replacement (IFR) Methods

In the indirect fitness approximation method, the original fitness function is used during the EA process while one or more components (typically variation operators) of the MOEA are assessed in the approximated fitness. The general idea is to produce several solutions and evaluate them in the metamodel in order to avoid real fitness function evaluations. These solutions are compared among them. This procedure is performed for a number of iterations. After a stop condition is reached, *n* best solutions are delivered to the parent approach. These solutions will be evaluated in the original problem. These techniques are known as informed operators [49, 50]. The approximated fitness can also be used to generate a random initial population in a similar manner. Using the approximated fitness indirectly is expected to keep the optimization towards the true Pareto front and at the same time to reduce the risk of convergence to a false optimum:

(i) Gaspar-Cunha et al. [46, 51] proposed using an inverse ANN as a local search strategy. The inverted ANN is constructed with some nondominated points taken from the previous generation. The input of the inverse ANN is a predefined objective value while the output is a prediction of the corresponding decision variables. This approach was applied to several benchmark problems with up to 30 variables and to a polymer extrusion real-world problem with four variables. According to the authors, a reduction of more than six hours of computational time was achieved.
(ii) Chafekar et al. [49] proposed the objective exchange genetic algorithm for design optimization (OEGADO). OEGADO executes several single-objective GAs concurrently (one GA for each objective). Each single-objective algorithm uses least-squares approximation to form the metamodel of its own objective. At equal intervals, each GA exchanges its own reduced model. In this algorithm, conventional operators such as initialization, mutation, and crossover are replaced by informed operators. The best individual based on this approximated fitness is selected to be evaluated in the real function and then it is added to the population. This approach was compared using four biobjective problems and two three-objective problems. However, the authors did not indicate the number of variables of each adopted test problem. The results showed that for simple biobjective problems the algorithm performed similarly to NSGA-II and the *ϵ*-MOEA [23]. However, for difficult biobjective problems, the OEGADO produced the best results.
(iii) Emmerich et al. [52] proposed a method based on an evolutionary strategy that uses a number of different predefined criteria (prescreening procedures) for multiobjective optimization, including the expected improvement and the probability of improvement. The improvement in this case is relative to the approximated Pareto set achieved so far (it is not a single value). Thus, the selected solutions will be those that increase the estimated hypervolume of the current nondominated set. In experiments, four different screening criteria on biobjective and three-objective problems with ten variables were compared. The results showed that the proposed approach outperformed NSGA-II in the adopted test functions.
(iv) Knowles [53] modified a Gaussian stochastic process model called the efficient global optimization (EGO) algorithm [54] in order to enable it to handle multiobjective problems. The resulting approach was named Pareto efficient global optimization (ParEGO). To build up a Pareto front, ParEGO uses a series of weighting vectors to convert the objective functions into a scalar. In each iteration, a new candidate solution is determined by (1) computing the expected improvement in a specific direction by the drawn weighting vector for that iteration and (2) searching a point that maximizes the expected improvement (a single-objective evolutionary algorithm is used for this issue). A suite of nine difficult but low-dimensional (up to eight variables) multiobjective limited ruggedness test functions were used to validate the algorithm. ParEGO generally outperformed NSGA-II on the tested functions, using both 100 and 250 function evaluations.
(v) Adra et al. [55] proposed using surrogate models with a local search strategy to improve the convergence of two MOEAs, NSGA-II and the strength Pareto evolutionary algorithm 2 (SPEA2). This approach requires as input the goal to be achieved (in the objective function space) and then it calculates, through the use of ANN, the values of the associated decision variables. In this work, the authors showed that the predicted solutions are close to the real values. They solved two biobjective problems taken from the Zizler-Deb-Thiele (ZDT) benchmark, ZDT1 and ZDT3, and showed a reduction of 75% in the number of generations needed to obtain a good representation of the Pareto front. However, the authors did not indicate the number of variables used for the comparison.
(vi) Georgopoulou and Giannakoglou [56] combined the SPEA2 with a local search operator. The proposed approach uses an inexact preevaluation approach (IPE) which starts after running SPEA2 for just a few generations on the exact evaluation model. All the evaluated solutions are stored in a database. In subsequent generations, local metamodels are constructed for each new offspring. The metamodels are used to reach an approximation of both the objective functions and the gradient to be used during a local search. The metamodel used is RBF network. This approach was validated using a single test function with 30 variables and two real-world problems: (1) the design of a combined cycle power plant and (2) the multipoint design of a compressor cascade airfoil.
(vii) Loshchilov et al. [57] proposed an approach that uses a global surrogate model in order to speed up the evolution progress of MOEA towards the true Pareto front. The surrogate model is specified by combining a one-class SVM to characterize the dominated points and an SVR to clamp the Pareto front on a single value. This surrogate model was intended to guide the search in the vicinity of the current Pareto set and to speed up the population movement towards the true Pareto set. This surrogate model was named the aggregated surrogate model (ASM). The authors proposed an informed operator that uses ASM called Pareto-SVM. The proposed operator was integrated into NSGA-II and into the multiobjective covariance matrix adaptation evolution strategy (MO-CMA-ES) in order to ascertain the improvement obtained with the use of the operator. The comparison was made on a set of biobjective test functions with up to 30 variables. The results showed that the proposed approach obtained a better convergence speed than the compared state-of-the-art algorithms.
(viii) Zhang et al. [58] proposed coupling MOEA/D with the EGO algorithm. The resulting approach was named MOEA/D-EGO. On each iteration, MOEA/D-EGO constructs a Gaussian process model for each subproblem based on the solutions obtained in the previous search. Then, the expected improvements of these subproblems are optimized simultaneously using MOEA/D to generate a set of candidate test points. A few of them are selected with respect to their expected improvement for real function evaluation. The proposed approach was compared with respect to ParEGO. Several test functions, with two to six objective functions and up to eight variables, were compared. The results show that MOEA/D-EGO outperformed ParEGO in most test functions.
(ix) Martínez and Coello Coello [59, 60] proposed a memetic algorithm of the non-gradient-based local search assisted by SVMs. The local search mechanism adopts a free-derivative mathematical programming technique that works in two phases. In the first phase, a set of solutions is obtained from the optimization of aggregating functions which are defined for a fixed number of weighted vectors. These solutions are used for the second phase as the initial solutions using a differential evolution (DE). The local search mechanism is used into the metamodel. Finally, the new solutions are incorporated in the MOEA using a Pareto ranking scheme. According to the authors, the memetic algorithm produced good results with only 1,000 evaluations in problems with ten and 30 decision variables. However, the authors did not evaluate the approach in problems with three or more objectives.
(x) Arias-Montaño et al. [61] proposed a surrogate-based MOEA to optimize airfoil aerodynamic designs. In this approach, the authors argued that the balance of exploration/exploitation can be achieved through the use of several surrogate models, in such a way that solutions selected from a highly accurate surrogate model will improve the exploitation, while solutions produced in a surrogate with low accuracy will help in the exploration. In this approach, the authors adopted *N* parallel surrogate models, each one optimized with the MODE-LD+SS [62]. In such a manner, one solution is extracted from each surrogate model. Therefore, *N* new ones are generated on each generation. To make the selection, first a set of weight vectors is defined. Next, from each surrogate model, the approach selects the solution that minimizes a Tchebycheff scalar function. These solutions are the new solutions in the MOEA. This approach was successfully employed to solve five multiobjective airfoil aerodynamic optimization problems with 12 variables. The results indicate that this approach can achieve a substantial reduction in the number of objective function evaluations.
(xi) Pilát and Neruda [63] proposed a method that can be plugged into any existing MOEA in order to improve its performance. On each generation, this approach executes the variation operators of the external algorithm; then an evolutionary algorithm is used to locally improve some of the individuals. Additionally, a local search phase is used. In this phase, a surrogate model is constructed to predict the distance of a particular individual to the current known Pareto front so that an aggregate surrogate model provides a single value which expresses the quality of the individual. Such a model is used as a fitness function of a single-objective evolutionary algorithm during the local search phase. This approach was incorporated into NSGA-II and the *ϵ*-indicator-based evolutionary algorithm (*ϵ*-IBEA) [25] and was tested on the ZDT test suite with 15 variables. According to the authors, this approach reduces (by one order of magnitude) the number of evaluations required for acceptable results. However, the authors also noticed that the method presented some problems in later phases of the evolution, leading to reducing the convergence to the true Pareto front. The authors also proposed similar approaches [64, 65]; however, the surrogate models were used in two different ways, during a local search and during a preselection phase. During the preselection phase, a surrogate model is constructed for each objective function in order to be used to predict the value of the objective functions. Only those individuals that are not dominated by any of their parents are then evaluated in the real objective function and considered to be selected for the next generation. The results obtained by this algorithm were compared with those obtained by NSGA-II and *ϵ*-IBEA in biobjective test functions of high dimensionality (30 variables). The authors showed that the number of required evaluations decreased significantly, in some cases by almost 50%. Additionally, the authors proposed a similar approach but using local surrogate models [66].
(xii) Bittner and Hahn [67] proposed a method to combine a multiobjective particle swarm optimization algorithm and a Kriging algorithm. In this approach, the expected growth of the dominated hypervolume is calculated for each particle. Then, the particles are sorted according to the expected growth. After that, only the highest ranked particles are selected to be assessed by the real objective function. Those particles with the worst results are spawned at new positions which are determined by a separate optimization that is based on the surrogate model. This approach was tested in a biobjective test function of ten variables. The results showed that the approach required only 400 evaluations in order to reach the true Pareto front. Additionally, the approach was used to design a ten-pole inner rotor PMSM using three objectives. This problem was solved with only 1200 exact evaluations. However, in this problem the approach was not compared with any other MOEA.

*Review*. Most of the existing works in this category have a local search phase where the metamodel is employed; the best solutions produced by such a local search are assessed in the real problem. Therefore, we can say that these approaches use metamodels for exploitation purposes, while the MOEA is employed to direct a coarse grain search. It is worth noting that the outer approach (the MOEA) always works exclusively with solutions evaluated in the real problem, thus avoiding a convergence to a false Pareto front.

In this category, it is possible to distinguish a more varied number of search engines. However, in this category it is also necessary to justify the selection of the surrogate model. Probably, a previous study of the selection of the surrogate model can improve the obtained results of the existing approaches. Moreover, some approaches in this classification depend exclusively on a particular surrogate model [53, 58].

Finally, although the previously reviewed works were not proposed as a single component of MOEA, we can modify them to be used as an element of any MOEA.

## 4. Discussion

Table 1 shows the main advantages and disadvantages of each class in the taxonomy used. Adittionally, Table 2 shows several characteristics of the reviewed approaches. We present below our main findings.

We have divided our reviewed approaches into two main classes, the direct fitness replacement (DFR) and the indirect fitness replacement (IFR). The former is divided into three subclasses, no evolution control (NEC), fixed evolution control (FEC), and adaptive evolution control (AEC).

Once the surrogate model is built, NEC approaches never use the original problem to evaluate a solution or to get an error feedback. To be successful, problems handled by these approaches must have a search space easy to surrogate. Therefore, these approaches are not generic but are developed for a specific problem. The 100% of the NEC approaches here reviewed solved exclusively engineering problems (they did not solve any synthetic problem). Also, the surrogate model approaches adopted by NEC proposals are response surface variants (three approaches are PRS based and three approaches are KRG based), and only one reviewed proposal performed a comparison of the surrogate model techniques. Additionally, NSGA-II was the preferred approach, since 66% of the proposals adopted it. Finally, the nature of NEC approaches makes it inviable to be used as an independent operator.

FEC is the most popular DFR-based subclass with nine proposals. However, it is also the one that solved the fewest engineering problems, with five (55%). Response surface is the most prominent metamodeling technique with four (44%, three PRS approaches and one KRG approach). RBF is the second most prominent surrogate model (three approaches or 33%). Similarly to NEC, most approaches from this class also incorporated NSGA-II as their preferred MOEA with five out of nine reviewed approaches (55%). However, only one proposal performed a comparison of metamodeling techniques, and only two approaches approximated the search space by regions.

The high degree of complexity of AEC makes it the less trendy DFR-based subclass with only three proposals. These proposals incorporated a GA-based MOEA and also approximate the whole search space. From these approaches, two solved engineering problems, and only one performed a comparison among surrogate models. It is worth noting that these approaches solved problems with 30 variables or more.

IFR approaches can be easily coupled into a different MOEA, since only one approach from this class could not be treated as an independent operator. Although IFR is very popular among researchers, only 42% of its approaches solved engineering problems. Similarly, all IFR methods' approaches were a viable option to reduce the number of function evaluations required to achieve good results of any MOEA.

19 of the 32 reviewed approaches solved engineering problems. KRG was incorporated in seven approaches, PRS was incorporated in five, RBF was incorporated in four, ANN was incorporated in 2, and GP was incorporated in one approach. This means that most engineering problems were solved using two approaches, response surface approaches (KRG and PRS) with 63% and ANN-based approaches (RBF and ANN) with 31%. Additionally, engineering problems usually use ad hoc approaches since 14 out of 19 proposals (73%) can not be used as independent operators. NSGA-II was presented in 36% of the proposals that solved engineering problems.

Unlike what one would expect, only six works performed comparison between metamodeling techniques, two of them adopted accuracy to assess the performance of the compared surrogate model techniques, and five works evaluated how the tuple (metamodeling technique, MOEA) behaved. Response surface was the metamodeling technique most widespread, since it was adopted by five out of the six approaches (three PRS and two KRG approaches). NSGA-II was the preferred MOEA with 83% of selection. Furthermore, 50% of these approaches approximate the search space by regions (LM), while the remaining approximate the whole search space (GM). Finally, only two approaches solved engineering problems.

PRS was presented in 4 out of 6 proposals that approximate the search space by regions (LM). This could indicate that local approximation does not require sophisticated approaches in order to achieve acceptable results.

## 5. Conclusions

In this paper, we have reviewed MOEAs that make use of surrogate models in order to reduce the real fitness function evaluations. The reviewed proposals were grouped according to their working styles.

Most of the reviewed approaches had not taken the accuracy into account. Also, they did not compare the metamodels in order to identify which approach was best suited for use. Moreover, when the approaches are intended to solve a specific problem, selecting the most appropriate metamodel can considerably improve the accuracy, thus improving the general performance.

Additionally, it can be observed that most of the existing works solved low-dimensional biobjective problems. Therefore, it is necessary to start focusing on problems with higher dimensionality in both spaces (i.e., objective and decision).

Moreover, most of the reviewed works use NSGA-II as the underlying MOEA. However, several efficient MOEAs can be easily adapted in order to replace the current approach. Also, we found that many of the proposed approaches lack a proper methodology for results comparison since the performances of the proposed approaches were not compared with respect to other multiobjective approaches.

Another important unexploited feature is the use of local surrogate models instead of global surrogate models [38, 66]. According to the results achieved by several authors, building local surrogate models on smaller regions may impact the accuracy of the model.

Moreover, although the use of external archives in multiobjective optimization has been a recurrent trend, it has been practically forgotten in current surrogate-based MOEAs.

Finally, another issue that certainly deserves attention is the need for theoretical foundations for surrogate-based MOEAs.

## Figures and Tables

**Table 1:** Advantages and disadvantages of each class in the taxonomy.

Class	Advantages	Disadvantages
*DFR*		
No evolution control	(i) Computationally efficient (ii) Good behavior in low-dimensional problems	(i) Requires an accurate surrogate model (ii) It can converge to a false optimum
Fixed evolution control	(i) It is capable of solving high-dimensional problems (ii) The surrogate model adapts during the optimization process	(i) It is necessary to define the parameter to alternate between the surrogate model and the real objective function
Adaptive evolution control	(i) Does not require defining the parameter to alternate between the surrogate model and the real objective function	(i) The automatic alternation is not easy to define

*IFR*	(i) Usually uses a local search phase to optimize the surrogate model (ii) The metamodel is used for exploitation purposes (iii) Avoid convergence to a false optimum	(i) It is most computationally expensive

**Table 2:** Summary of features of the reviewed works.

Reference	Year	CLS	Op	LM/GM	MC	AM	MOEA	ENG_(vars,objs)_	SYNT_(vars,objs)_
Choi et al. [14]	2004	NEC	*✕*	GM	*✕*	KRG	NSGA-II	✓ (17, 2)	*✕*
Lian and Liou [16]	2005	NEC	*✕*	GM	*✕*	PRS	GA	✓ (8, 2)	*✕*
Gonzalez et al. [18]	2006	NEC	*✕*	GM	*✕*	KRG	HAPEA	✓ (53, 2)	*✕*
Goel et al. [20]	2007	NEC	*✕*	GM	*✕*	PRS	NSGA-II	✓ (4, 4)	*✕*
Liao et al. [21]	2008	NEC	*✕*	GM	*✕*	PRS	NSGA-II	✓ (5, 3)	*✕*
Husain and Kim [22]	2010	NEC	*✕*	GM	✓^a,s^	KRG	NSGA-II	✓ (3, 2)	*✕*
Nain and Deb [34]	2002	FEC	*✕*	GM	*✕*	ANN	NSGA-II	*✕*	✓ (39, 2)
D'Angelo and Minisci [35]	2005	FEC	*✕*	GM	*✕*	KRG	MOPED	✓ (5, 2)	*✕*
Voutchkov and Keane [37]	2006	FEC	*✕*	GM	✓^s^	KRG	NSGA-II	*✕*	✓ (10, 2)
Isaacs et al. [38]	2007	FEC	*✕*	LM	*✕*	RBF	NSGA-II	*✕*	✓ (10, 2)
Todoroki and Sekishiro [39]	2008	FEC	*✕*	GM	*✕*	KRG	MOGA	✓ (7, 2)	*✕*
Liu et al. [41]	2008	FEC	*✕*	LM	*✕*	PRS	MOGA	✓ (3, 2)	✓ (3, 2)
Fonseca et al. [42]	2010	FEC	*✕*	GM	*✕*	FIT^1^	NSGA-II	*✕*	✓ (30, 3)
Zapotecas Martínez and Coello Coello [43]	2013	FEC	*✕*	GM	*✕*	RBF	MOEA/D	✓ (12, 2)	✓ (30, 2)
Stander [45]	2013	FEC	*✕*	GM	*✕*	RBF	NSGA-II	✓ (7, 2)	✓ (30, 2)
Gaspar-Cunha and Vieira [46]	2005	AEC	*✕*	GM	*✕*	ANN	GA	✓ (4, 2)	✓ (30, 2)
Sreekanth and Datta [47]	2010	AEC	*✕*	GM	✓^a^	GP	NSGA-II	✓ (33, 2)	*✕*
Rosales-Perez et al. [48]	2013	AEC	*✕*	GM	*✕*	SVM	GA	*✕*	✓ (30, 2)
Gaspar-Cunha et al. [46, 51]	2005	IFR	*✕*	GM	*✕*	ANN	GA	✓ (4, 2)	✓ (30, 2)
Chafekar et al. [49]	2005	IFR	✓	GM	*✕*	PRS	OEGADO	✓ (6, 2)	✓ (30, 3)
Emmerich et al. [52]	2006	IFR	✓	GM	*✕*	KRG	NSGA-II	✓ (5, 3)	✓ (10, 2)
Knowles [53]	2006	IFR	✓	GM	*✕*	KRG	EGO	*✕*	✓ (10, 3)
Adra et al. [55]	2007	IFR	✓	GM	*✕*	ANN	NSGA-II, SPEA2	*✕*	✓ (30, 2)
Georgopoulou and Giannakoglou [56]	2009	IFR	✓	LM	*✕*	RBF	SPEA2	✓ (13, 2) (20, 2)	✓ (30, 2)
Loshchilov et al. [57]	2010	IFR	✓	GM	*✕*	CUST^2^	NSGA-II, MO-CMA-ES	*✕*	✓ (30, 2)
Zhang et al. [58]	2010	IFR	✓	GM	*✕*	KRG	MOEA/D	*✕*	✓ (8, 2) (6, 3)
Zapotecas Martínez and Coello Coello [59, 60]	2010	IFR	✓	GM	*✕*	SVM	DE	*✕*	✓ (30, 2)
Arias-Montaño et al. [61]	2012	IFR	✓	GM	*✕*	RBF^3^	MODE-LD+SS	✓ (12, 3)	*✕*
Pilát and Neruda [63, 68]	2011	IFR	✓	LM	✓^s^	PRS	NSGA-II, ϵ-IBEA	*✕*	✓ (15, 2)
Pilát and Neruda [64, 65]	2012	IFR	✓	LM	✓^s^	PRS, SVR	SBMO-ES	*✕*	✓ (30, 2)
Pilát and Neruda [66]	2013	IFR	✓	LM	✓^s^	PRS, SVM, ANN	NSGA-II, ϵ-IBEA	*✕*	✓ (30, 2) (20, 15)
Bittner and Hahn [67]	2013	IFR	✓	GM	*✕*	KRG	PSO	✓ (11, 3)	✓ (10, 2)

CLS: classification. Op: the proposal can be incorporated as an independent operator.

LM/GM: local or global surrogate model. AM: adopted surrogate model approach.

MC: surrogate model comparison. ENG/SYNT: engineering or synthetic problems.

^a^Accuracy. ^s^The Suitability of the surrogate model technique to be coupled into MOEA.

^1^Fitness inheritance and fitness imitation.

^2^Customized metamodeling technique.

^3^RBF with multiple kernels.
